# RETRACTION: Apelin‐13 Is an Early Promoter of Cytoskeleton and Tight Junction in Diabetic Macular Edema via PI‐3K/Akt and MAPK/Erk Signaling Pathways

**DOI:** 10.1155/bmri/9757309

**Published:** 2026-03-31

**Authors:** BioMed Research International

RETRACTION: Y. Li, Y‐J. Bai, Y‐R. Jiang, W‐Z. Yu, X. Shi, L. Chen, J. Feng, G‐B. Sun, “Apelin‐13 Is an Early Promoter of Cytoskeleton and Tight Junction in Diabetic Macular Edema via PI‐3K/Akt and MAPK/Erk Signaling Pathways,” *BioMed Research International*, 2018, 3242574, https://doi.org/10.1155/2018/3242574

The above article, published online on 04 April 2018 in Wiley Online Library (wileyonlinelibrary.com), has been retracted by agreement between the journal Editor‐in‐Chief, Dr. Yoel A. Klug; and John Wiley & Sons Ltd. The retraction has been agreed due to multiple errors in the figures. Initially the authors requested to correct Figure 2, however subsequent investigation identified additional concerns with Figures 3, 6 and 8:

• In Figure 2d, the Nuclear/NG + A 100 ng/ml panel appears similar to the Merge/NG + A 1000 ng/ml panel
• In Figure 3, the Actin blots shown in 3h appear similar to the Src blots shown in 3j when resized
• In Figure 6, the Actin blots shown in 3a, 3c and 3d appear similar
• In Figure 8, the Actin blots shown in 8a, 8b and 8d appear similar

After assessment of the concerns and author’s response by the Chief Editor, the results and conclusions are no longer deemed reliable, and the article is retracted.

The authors agree to the retraction.

